# Penile anthropometry in adolescents and adults systemic lupus erythematosus

**DOI:** 10.1186/1546-0096-9-S1-P256

**Published:** 2011-09-14

**Authors:** Ana P Vecchi, Eduardo F Borba, Eloísa Bonfá, Marcelo Cocuzza, Patrícia Pieri, Chong A Kim, Clovis A Silva

**Affiliations:** 1 Rheumatology Division, Faculdade de Medicina da Universidade de São Paulo, São Paulo – Brazil; 2 Urology Department, Faculdade de Medicina da Universidade de São Paulo, São Paulo – Brazil; 3 Pediatric Rheumatology Unit, Faculdade de Medicina da Universidade de São Paulo, São Paulo – Brazil

## Aim

The objective of this study was to evaluate penile anthropometry in systemic lupus erythematosus (SLE) patients and controls.

## Methods

Twenty-five consecutive SLE patients were assessed by urological examination, sexual function, testicular ultrasound, hormones, sperm analysis, genetic analysis, clinical features and treatment. The control group included 25 healthy age-matched healthy men.

## Results

SLE patients had a lower median penis length and circumference [8(7.5-10) vs. 10(8-13) cm, p=0.0001; 8(7-10) vs. 10(7-11) cm, p=0.001; respectively], lower median testicular volume by right and left Prader [15(10-25) vs. 20(12-25) ml, p=0.003; 15(10-25) vs. 20(12-25) ml, p=0.006; respectively], higher median of FSH [5.8(2.1-25) vs. 3.3(1.9-9) IU/l, p=0.002] and lower morning total testosterone levels (28% vs. 0%, p=0.009) compared to controls. In spite of that, erectile dysfunction was not observed in patients or controls. Analyses of lupus patients revealed that the median penis circumference was lower in patients with disease onset before first ejaculation compared to those that began after first ejaculation [7.8(7-10) vs. 9.0(7.5-10) cm, p=0.026]. No differences were observed in the median penile anthropometry regarding sexual dysfunction (p=0.610), lower total testosterone levels (p=0.662), oligo/azoospermia (p=0.705), SLEDAI ≥ 4 (p=0.562), SLICC/ACR Damage Index ≥ 1 (p=0.478), prednisone cumulative dose (p=0.789) and intravenous cyclophosphamide therapy (p=0.754). KlinefelterÂ´s syndrome (46XY/47XXY) was diagnosed in one (4%) SLE patient with decreased penile size whereas Y-chromosomal microdelections was absent in all of them.

## Conclusion

We have identified reduced penile dimensions in SLE patients with no deleterious effect in erectile function. Disease onset before first ejaculation seems to affect penis development in pre-pubertal lupus.

